# Sustained activation of sphingomyelin synthase by 2-hydroxyoleic acid induces sphingolipidosis in tumor cells^1^[S]

**DOI:** 10.1194/jlr.M036749

**Published:** 2013-05

**Authors:** Maria Laura Martin, Gerhard Liebisch, Stefan Lehneis, Gerd Schmitz, María Alonso-Sande, Joan Bestard-Escalas, Daniel H. Lopez, José Manuel García-Verdugo, Mario Soriano-Navarro, Xavier Busquets, Pablo V. Escribá, Gwendolyn Barceló-Coblijn

**Affiliations:** * Laboratory of Molecular Cell Biomedicine, Department of Biology, University Institute for Research into Health Sciences (IUNICS), University of the Balearic Islands, E-07122 Palma, Balearic Islands, Spain; † Institute for Clinical Chemistry and Laboratory Medicine, University of Regensburg, 93042 Regensburg, Germany; and; § Laboratorio de Morfología Celular, Unidad Mixta Centre d'Investigació Príncep Felipe-Universitat de València Estudis Generals (CIPF-UVEG), Centro de Investigación Biomédica en Red, Enfermedades Neurodegenerativas (CIBERNED), 46013 Valencia, Spain

**Keywords:** antitumor drug, sphingolipid metabolism, mass spectroscopy

## Abstract

The mechanism of action of 2-hydroxyoleic acid (2OHOA), a potent antitumor drug, involves the rapid and specific activation of sphingomyelin synthase (SMS), leading to a 4-fold increase in SM mass in tumor cells. In the present study, we investigated the source of the ceramides required to sustain this dramatic increase in SM. Through radioactive and fluorescent labeling, we demonstrated that sphingolipid metabolism was altered by a 24 h exposure to 2OHOA, and we observed a consistent increase in the number of lysosomes and the presence of unidentified storage materials in treated cells. Mass spectroscopy revealed that different sphingolipid classes accumulated in human glioma U118 cells after exposure to 2OHOA, demonstrating a specific effect on C16-, C20-, and C22-containing sphingolipids. Based on these findings, we propose that the demand for ceramides required to sustain the SMS activation (ca. 200-fold higher than the basal level) profoundly modifies both sphingolipid and phospholipid metabolism. As the treatment is prolonged, tumor cells fail to adequately metabolize sphingolipids, leading to a situation resembling sphingolipidosis, whereby cell viability is compromised.

The mechanism of action of 2-hydroxyoleic acid (2OHOA), a potent antitumor drug, involves the specific and sequential induction of cell cycle arrest (1), cell differentiation (2), and cell death in human cancer cells (1–6). Based on its high efficacy and low toxicity compared with other existing chemotherapy agents, the European Medicines Agency has acknowledged the potential benefits of 2OHOA and has designated this molecule as an orphan drug for the treatment of glioma (7). In the present study, we investigated the mechanism of action of 2OHOA to further clarify the features underlying its unique combination of high efficacy, low toxicity, and specificity.

By activating sphingomyelin synthase (SMS) isozymes, 2OHOA increases SM mass in a rapid and highly specific manner (6). Indeed, the effect of 2OHOA on the cell cycle is partially reversed by SMS inhibition, suggesting that its activation is critical in its mechanism of action. SMS proteins catalyze the transfer of a phosphocholine moiety to the primary hydroxyl group of ceramide to form SM and 1,2-diacylglycerol (1,2-DAG) (8). However, the source of the ceramides required to maintain the sustained synthesis of SM induced by 2OHOA has yet to be defined. The increase in sphingosine (Sph) mass following 2OHOA treatment previously described already suggests that the salvage pathway is activated (6, 9). However, ceramides can be also synthesized via two alternative mechanisms: the “de novo” pathway, through the condensation of L-serine and palmitic acid; and the SM cycle (10).

Accordingly, we examined the effect of 2OHOA on ceramide metabolism in U118 human brain cancer cells, showing that 2OHOA activated the pathway of de novo ceramide synthesis and the recycling pathway. Furthermore, exposure to 2OHOA induced a profound alteration in sphingolipid metabolism, resulting in the accumulation of SM, ceramide, and hexosylceramide (HexCer) species. This sphingolipid accumulation was associated with an increase in lysosomal number and the formation of unidentified intracellular structures that were probably composed of the sphingolipids accumulated. Based on these results, we propose that the rapid and sustained activation of SMS generates an unusual demand for ceramide, provoking a profound shift in sphingolipid metabolism in order to maximize ceramide production. When exposure to 2OHOA is prolonged (48–72 h), tumor cells fail to fulfill this high metabolic requirement, resulting in a situation that resembles sphingolipidosis, in which cell viability is compromised. At this point, tumor cells trigger the activation of different pathways, such as cell cycle arrest and differentiation or cell apoptosis, producing a decrease in tumor cell number. These results complement our previous findings and allow a temporal sequence of events to be established that accounts for the antitumor effects of 2OHOA.

## MATERIALS AND METHODS

### Cell culture

Human glioma cells (U118) and human lung adenocarcinoma cells (A549) were obtained from the American Type Culture Collection (Manassas, VA) and maintained as described previously (4).

### Lipids

The 2OHOA (99.7%) compound (Good Manufacturing Practice quality) was obtained from Avanti Polar Lipids. NBD-C6-ceramide (NBD-Cer) and NBD-C6-sphingomyelin (NBD-SM) were purchased from Invitrogen (Barcelona, Spain). NBD-glucosylceramide (NBD-GluCer) was purchased from Larodan (Malmö, Sweden), and NBD-phosphatidylethanolamine (NBD-PE) and NBD-phosphatidylcholine (NBD-PC) were obtained from Avanti Polar Lipids (AL). Spectroscopic-grade organic solvents for the lipid and probe solutions were purchased from Merck (Darmstadt, Germany).

### Lipid analysis by mass spectroscopy

Lipid extraction and mass spectrometry-based targeted lipid analysis was performed as described previously (11–16), in the presence of isotopic labeled lipids or nonnaturally occurring lipid species (as internal standards). Briefly, cell pellets were lysed in 0.1% SDS and sonicated, and then lipids were extracted from aliquots corresponding to 100 µg total protein (BCA assay). Lipids were quantified by electrospray ionization tandem mass spectrometry (ESI-MS/MS) in positive ion mode. Samples were quantified by direct flow injection analysis using the analytical setup described by Liebisch et al. (16). Sphingosine-based ceramides (Cer) were analyzed using a fragment ion of *m/z* 264 (15). For each lipid class two, nonnaturally occurring internal standards were added and quantification was achieved by calibration lines generated by addition of naturally occurring lipid species to the respective sample matrix. Liquid chromatography coupled to MS/MS (LC-MS/MS) was used to quantify HexCer, lactosylceramides (LacCer), sphingoid bases, and sphingosylphosphorylcholine (SPC) (14) as well as lysophospholipids, sphingosine-1-phosphate, and lysophosphatidic acid (13). Deisotoping and data analysis for all lipid classes were performed by self-programmed Excel macros according to the principles described previously (14, 16).

### Lipid analysis by TLC

After extraction with n-hexane:2-propanol (3:2, by vol) (17, 18), individual phospholipid classes were separated by TLC, and the amount of protein was measured as described previously (6, 19, 20).

### Analysis of the effect of 2OHOA on sphingolipid metabolism

Control and treated (200 µM, 24 h) U118 and A549 cells were incubated with NDB-C6-Cer, NDB-C6-GluCer, NDB-C6-SM, NDB-C6-PE, and NDB-C6-PC (3 μM) for 4 h prior to lipid extraction. After lipid extraction, NBD-C6-phospholipids were separated by HPTLC as described above, and the fluorescent lipids were visualized on a Bio-Rad Molecular Imager FX and quantified using Quantity One software (Bio-Rad).

### Metabolic labeling of cells to measure de novo [^3^H]ceramide synthesis

Control and treated (200 µM, 6 or 24 h) U118 cells were pulse labeled with [^3^H]palmitic acid (0.30 µCi/ml) for 5 min, and then total cell lipids were extracted and separated by TLC as described previously (21). The plates were dried, and the side with the standards was sprayed with a solution of 8% (w/v) H_3_PO_4_ containing 10% (w/v) CuSO_4_ before they were dried and charred over a heater to develop the nonradioactive standard bands. The area corresponding to each lipid was scraped off, and the radioactivity was measured by liquid scintillation counting. The levels of [^3^H]ceramide produced were normalized to the cellular protein content.

### Inhibition experiments

U118 cells were incubated with D609 for 16 h (200 μM) and 2OHOA (200 μM) was added 1 h after the addition of D609 (Tocris Bioscience, UK). After the incubation period, cell pellets were lysed in 0.1% SDS and sonicated. Lipids were extracted from aliquots corresponding to 100 µg total protein and analyzed by MS as previously described.

### Immunofluorescence labeling of lysosomes with LysoSensor

U118 cells were plated at a density of 1.1·× 10^4^ cell/cm^2^ on Chambered Coverglass (Lab-TekTM II, Thermo Fisher Scientific) as indicated above, in the presence or absence of 2OHOA (200 µM, 48 h). After treatment, the cells were incubated for 1 h with 1 µM LysoSensor Green DND-189 probe pH Indicator (pH 4.5–6; Invitrogen), with Hoechst (trihydrochloride trihidrate, 40 µg/ml; Invitrogen) added for the last 5 min. Stained samples were visualized on a Nikon Eclipse TE2000-S fluorescence microscope at 40× magnification.

### Electron microscopy

Cells were seeded at 1.1 × 10^4^ cell/cm^2^ in 4-well Lab-Tek chamber slides (Nalge Nunc International, Naperville, IL) as indicated above, and they were maintained in the presence or absence of 2OHOA (200 µM) for 48 or 72 h. The cells were postfixed in 2% OsO_4_ for 1 h at room temperature and stained with 2% uranyl acetate (in 70% ethanol) in darkness for 2 h at 4°C. Finally, the cells were rinsed in sodium phosphate buffer (0.1 M, pH 7.2), dehydrated in ethanol, and infiltrated overnight with Araldite (Durcupan; Fluka, Buchs SG, Switzerland). Following polymerization, embedded cultures were detached from the chamber slide and glued to Araldite blocks. Serial semi-thin (1.5 µm) sections were cut with an Ultracut UC-6 (Leica, Heidelberg, Germany), mounted onto slides, and stained with 1% toluidine blue. Selected semi-thin sections were glued (Super Glue, Loctite) to araldite blocks and detached from the glass slide by repeated freezing (in liquid nitrogen) and thawing. Ultrathin (0.06–0.09 µm) ultracut sections were obtained and stained with lead citrate. Finally, photomicrographs were obtained by transmission electron microscopy (FEI Tecnai G2 Spirit Biotwin) using a digital camera (Morada, Soft Imaging System, Olympus).

### Statistics

Statistical analyses were performed using GraphPad Prism 4.01 (GraphPad Software Inc., San Diego, CA). Unless otherwise indicated, data are expressed as the mean ± SEM of at least three independent experiments (n = 3). The statistical significance of the mean difference was determined using the Student *t*-test. Asterisks indicate a significant effect of treatment compared with controls: *P < 0.05; **P < 0.01; ***P < 0.001.

## RESULTS

### Sphingolipid mass increases in U118 cells after long-term exposure to 2OHOA

**Fig. 1.**
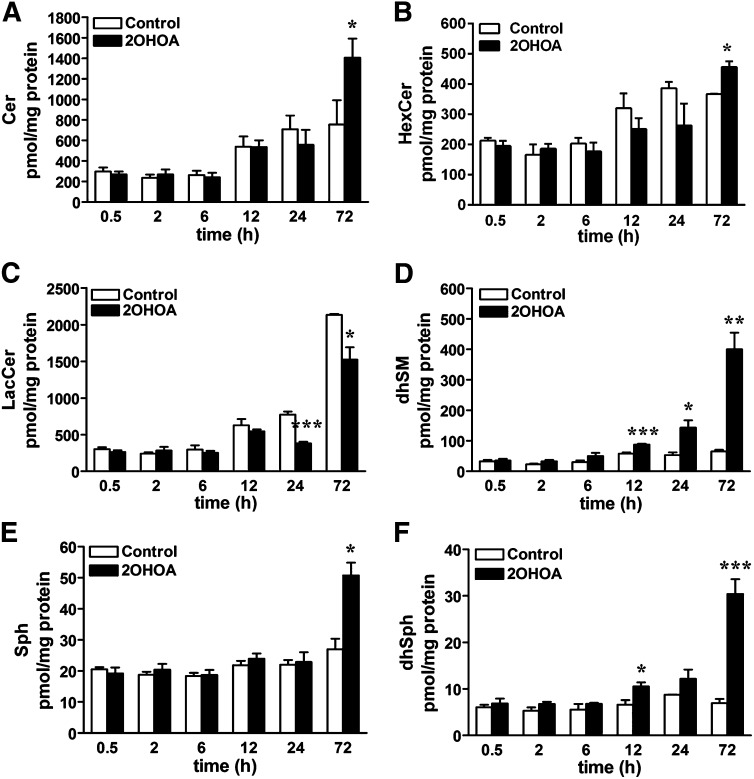
2OHOA treatment alters the sphingolipid composition in U118 cells. After U118 cells were exposed to 2OHOA for different times (200 µM, 0.5–72 h), the lipids were extracted and analyzed by mass spectrometry. (A) Total ceramide mass, (B) total HexCer mass, (C) total LacCer mass, (D) total dhSM mass, (E) Sph mass, and (F) dhSph mass. The values represent the mean ± SEM (n = 3–4). Asterisks (*) indicate significant effect of the treatment compared with controls: *P < 0.05, **P < 0.01, and ***P < 0.001.

Given the rapid effect of 2OHOA on SMS and the subsequent accumulation of SM (6), we used mass spectrometry to investigate the impact of 2OHOA (200 μM) on the rest of sphingolipids over a wide range of time points (0.5–72 h). Unexpectedly, no major changes in ceramide, HexCer, LacCer, Sph, dihydrosphingosine (dhSph), or dihydrosphingomyelin (dhSM) accumulation were detected following short exposures to 2OHOA (0.5–24 h; **Fig. 1**). The lack of changes does not exclude the possibility that the sphingolipid metabolism is not affected at that time. We addressed this point by using both radiolabeled and NBD-labeled substrates (see below). In addition, the lipidomic analysis showed that all these lipids, except LacCer, were increased after a 72 h exposure (**Table 1** and Fig. 1). Thus, the mass of ceramide, HexCer, and dhSM increased 1.9-, 1.2- and 4.6-fold, respectively, and similarly, Sph and dhSph mass increased 1.7- and 4.4-fold, respectively, while that of LacCer decreased 28.6% in treated cells. Free dhSph is mostly generated by de novo sphingolipid biosynthesis, whereas free Sph (the product of hydrolysis of complex sphingolipids) appears to be derived exclusively from the turnover of complex sphingolipids (9). Hence, these results are the first evidence indicating that both de novo ceramide synthesis (via dhSph accumulation) and the salvage pathway (via Sph accumulation) activation occurred after exposure to 2OHOA.

**TABLE 1.** Effect of 2OHOA treatment on the mass of different sphingolipid classes after 72 h of treatment

Sphingolipid Class (pmol/mg protein)	Control	Treated
Ceramides	755.7 ± 236.6	1406 ± 187.1*
Hexosylceramide	367.1 ± 1.0	456.5 ± 19.3*
Lactosylceramide	2124 ± 11.8	1525 ± 165.4*
Sphingosine	27.0 ± 3.4	50.8 ± 4.1**
Sphinganine	6.9 ± 0.9	30.4 ± 3.2**
Dihydrosphingomyelin	86.7 ± 21.96	400.0 ± 54.9**

After exposing U118 cells to 2OHOA for 72 h (200 µM), lipids were extracted and analyzed by mass spectrometry. The values represent the mean ± SEM (n = 3–4). Asterisks (*) indicate a significant effect of treatment compared with controls: *P < 0.05, **P < 0.01, and ***P < 0.001.

**Fig. 2.**
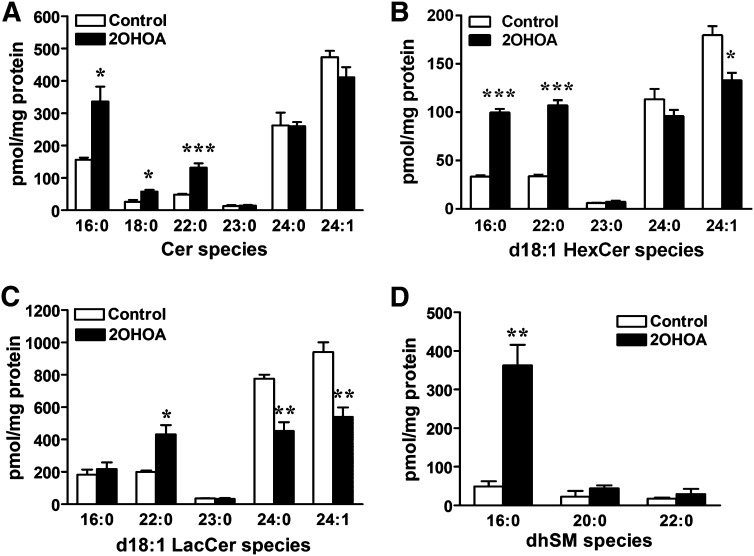
2OHOA modulates the profile of sphingolipid molecular species. After U118 cells were exposed to 2OHOA (200 µM, 72 h), lipids were extracted and analyzed by mass spectrometry. (A) Ceramide species mass; (B) HexCer species mass, (C) LacCer species mass, and (D) dhSM species mass. Values represent the mean ± SEM (*n* = 3). Asterisks (*) indicate a significant effect of treatment compared with controls: *P < 0.05, **P < 0.01, and ***P < 0.001.

MS analysis enabled the molecular species of each lipid class to be characterized in detail, which revealed a profound remodeling of the sphingolipid fatty acid composition following 2OHOA treatment (**Fig. 2**). The most consistent change among sphingolipids was the increase in C16- and C22-containing sphingolipid species, and after 72 h of treatment, the mass of C16-ceramide, C16-HexCer and C16-dhSM increased 2.2-, 3.0-, and 7.8-fold, respectively. Likewise, C22-ceramide, C22-HexCer and C22-LacCer increased 5.4-, 3.2-, and 2.2-fold, respectively. Interestingly, the mass of C24-sphingolipid species (24:0 and 24:1), which account for the 80% of total fatty acids, was either unaffected (in ceramides) or diminished (in HexCer and LacCer) following 2OHOA treatment. As a first approach to understand the specific effect on sphingolipid fatty acid composition, we investigated whether the treatment affected CerS mRNA expression levels (supplementary Fig. I). However, despite the large increase in C16- and C22-containing sphingolipids, no significant differences in CerS5 and CerS1 mRNA expression were observed in 2OHOA-treated cells and controls (200 μM, 72 h; supplementary Fig. IB).

### 2OHOA treatment profoundly modifies sphingolipid and phospholipid metabolism

**Fig. 3.**
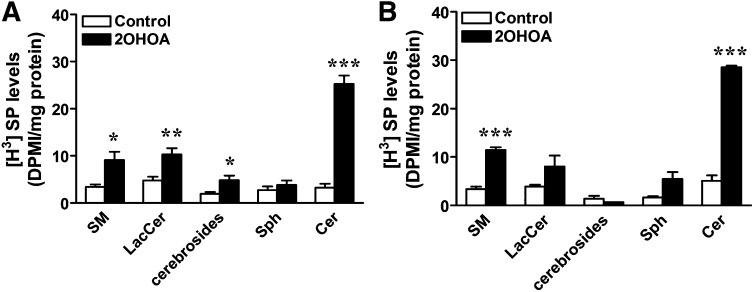
2OHOA induces de novo ceramide synthesis. U118 cells exposed to 2OHOA for 6 h (A) and 24 h (B) were pulse-labeled with [^3^H]palmitic acid for 5 min before the radioactive lipids were separated by TLC and quantified by liquid scintillation counting. HexCer includes GluCer+GalCer, [^3^H]SP and [^3^H]sphingolipids. Values represent the mean ± SEM (n = 3). Asterisks (*) indicate a significant effect of the treatment compared with controls: *P < 0.05, **P < 0.01, and ***P < 0.001.

**Fig. 4.**
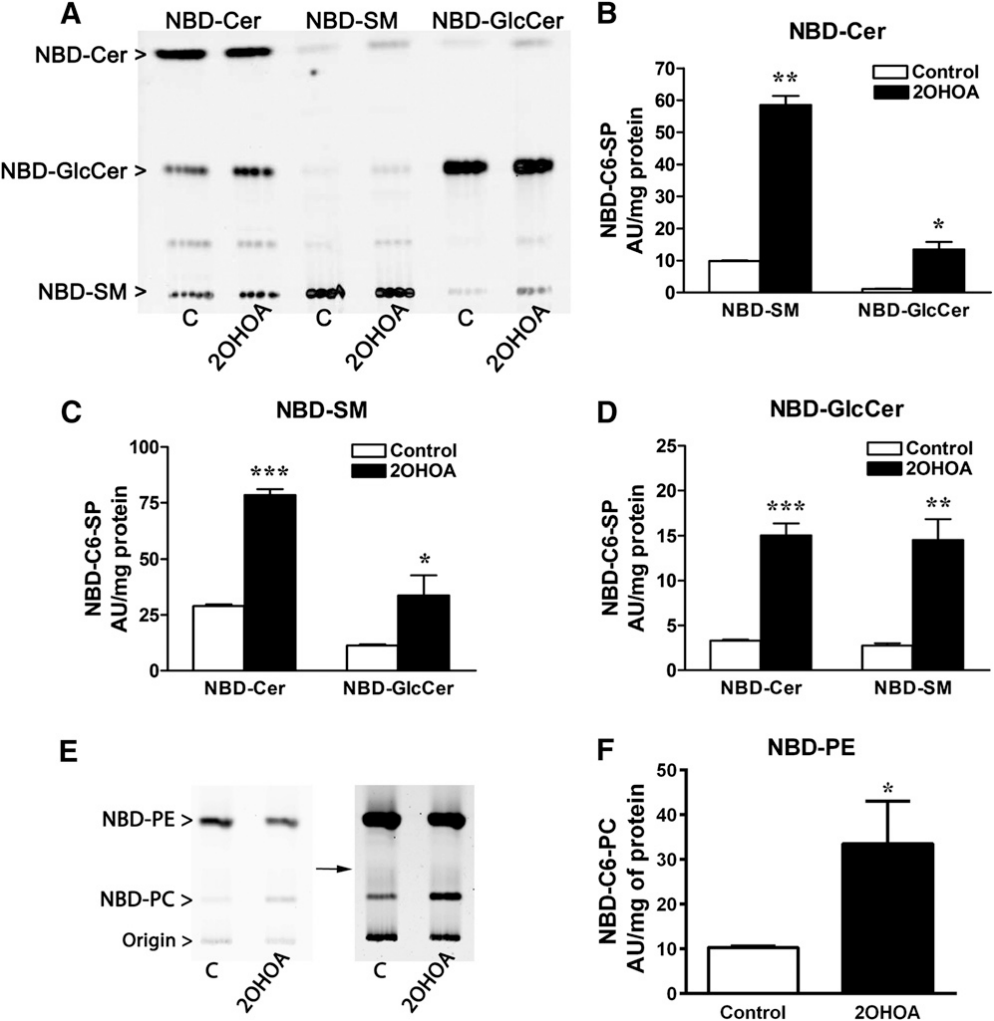
2OHOA alters the turnover of sphingolipids and phospholipids in U118 cells. (A) Representative TLC of lipid extracts from control and 2OHOA-treated cells (200 µM, 24 h) incubated with NBD-Cer (B), NBD-SM (C), or NBD-GlcCer (D: 3 µM, 3 h), from which lipids were extracted and analyzed by TLC. NBD-C6-SP, NBD-C6-sphingolipids. (E) Representative TLC of lipid extracts from control and 2OHOA-treated cells (200 μ, 24 h) incubated with NBD-C6-PE for 3 h, and from which lipids were extracted and analyzed by TLC. Separation was achieved using a two-solvent system (21). The image contrast was saturated to better visualize the minor bands (image on right side of the panel indicated with an arrow). AU, arbitrary units. The values represent the mean ± SEM (n = 3). Asterisks (*) indicate a significant effect of treatment compared with controls: *P < 0.05, **P < 0.01, and ***P < 0.001.

Based on the indirect evidence suggesting that both de novo synthesis and the salvage pathway are activated by 2OHOA, we further analyzed the effect of 2OHOA on these pathways in U118 cells. Activation of the de novo pathway was examined specifically by pulse-labeling with radioactive palmitate, the substrate of serine palmitoyltransferase (SPT) that is the first enzyme in this pathway (9). Cells were exposed to 2OHOA for 6 or 24 h and then pulse-labeled with [^3^H]palmitic acid for 5 min prior to lipid extraction. TLC analysis revealed a 7.8- and 5.6-fold increase in [^3^H]ceramide content following exposure to 2OHOA for 6 and 24 h, respectively (**Fig. 3**), confirming the activation of the de novo synthesis pathway. As expected, [^3^H]SM content increased after 2OHOA treatment (2.7-fold after 6 h and 3.4-fold after 24 h). Finally, the increases observed in the levels of [^3^H]LacCer and [^3^H]HexCer provided further evidence of a general modification of the sphingolipid metabolism. This was analyzed by incubating control and 2OHOA-treated (200 µM, 24 h) cells with the following NBD-C6 sphingolipid analogs (3 µM, 3 h): NBD-Cer, NBD-SM, and NBD-C6-GlcCer (**Fig. 4A–D**). Incubation with NBD-Cer or NBD-SM resulted in the accumulation of NBD-GlcCer (5.9- and 11.6-fold, respectively). Similarly, when cells were incubated with NBD-SM, we observed increased accumulation of NBD-Cer (2.7-fold) and NBD-GlcCer (3.0-fold). Finally, after exposure to NBD-GlcCer, the formation of NBD-Cer and NBD-SM increased 4.6- and 5.3-fold, respectively. Importantly, similar results were obtained in A549 cells (supplementary Fig. II). These results further confirm that both sphingolipid synthetic and degrading pathways are altered by exposing cells to 2OHOA for 24 h.

Collectively, our experiments using radioactive- and fluorescent-labeled lipids provide further evidence that de novo synthesis ([^3^H]palmitic acid labeling experiment), the salvage pathway (GluCer-NBD-labeling experiment) and the SM cycle (SM-NBD-labeling experiment) are activated after treatment with 2OHOA. Interestingly, the incubation of U118 cells with NBD-PE resulted in an unexpected increase in the formation of NBD-PC) (4-fold; Fig. 4E). The strong demand for PC as the second necessary substrate used by SMS along with ceramide may account for the activation of this pathway. In addition, this result may explain the significant decrease in PE mass previously described (6). Thus, the decrease in PE and PC masses (60 and 73 nmol/mg protein, respectively) correlated approximately with the increase in SM mass (151 nmol/mg protein; see Table 1 in Ref. 6).

**Fig. 5.**
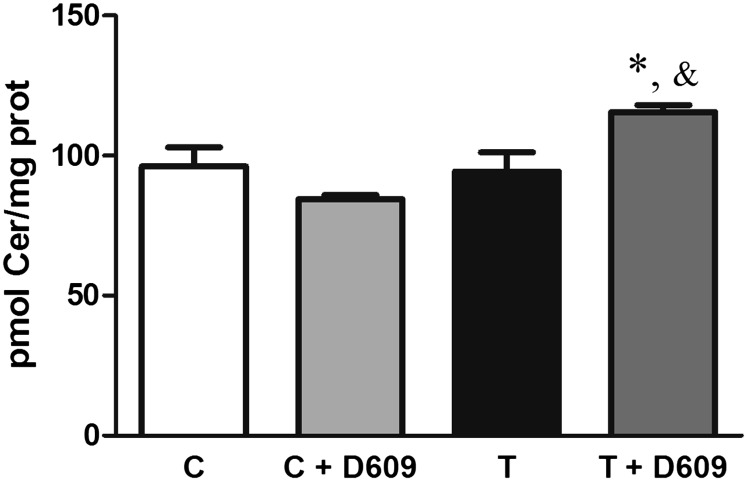
Effect on ceramide mass of the SMS inhibition by D609 in U118 cells. Inhibition of SMS by D609 in the presence of 2OHOA induced a minor accumulation of ceramides. The values represent the mean ± SEM (n = 3) and the asterisks (*) indicate a significant effect of treatment compared with controls: *P < 0.05. In this case, and indicates a significant effect of the inhibition compared with the treatment (*P* < 0.05).

Taking into account the avid consumption of ceramides by SMS and that the amount present in the cell was approximately two orders of magnitude lower than the amount of SM, our hypothesis was that cells try to compensate this severe unbalance by activating ceramide-generating pathways. So far we demonstrated that the de novo, the salvage, and the SMase cycles are activated. To test that the driving force leading cells to activate them is the reestablishment of the chemical equilibrium rather than a direct effect of 2OHOA on any of the enzymes involved in those pathways, we inhibited SMS using D609 (potassium tricyclodecan-9-yl xanthate) (22). If, in addition to SMS, any enzyme of the ceramide-producing pathways would be also activated by 2OHOA, then a large accumulation of ceramides would be expected. On the contrary, if only SMS is directly activated, then the increase would be lower. As shown in **Fig. 5**, the results supported our hypothesis. Despite the lack of accumulation of ceramides in the positive control (C+D609), the 30% decrease in SM mass observed after inhibition (5.8 ± 0.4 versus 4.1 ± 0.5 nmol/mg protein in the absence or presence of D609, respectively) indicated that SMS was indeed inhibited.

### Prolonged treatment with 2OHOA induces sphingolipidosis in tumor cells

**Fig. 6.**
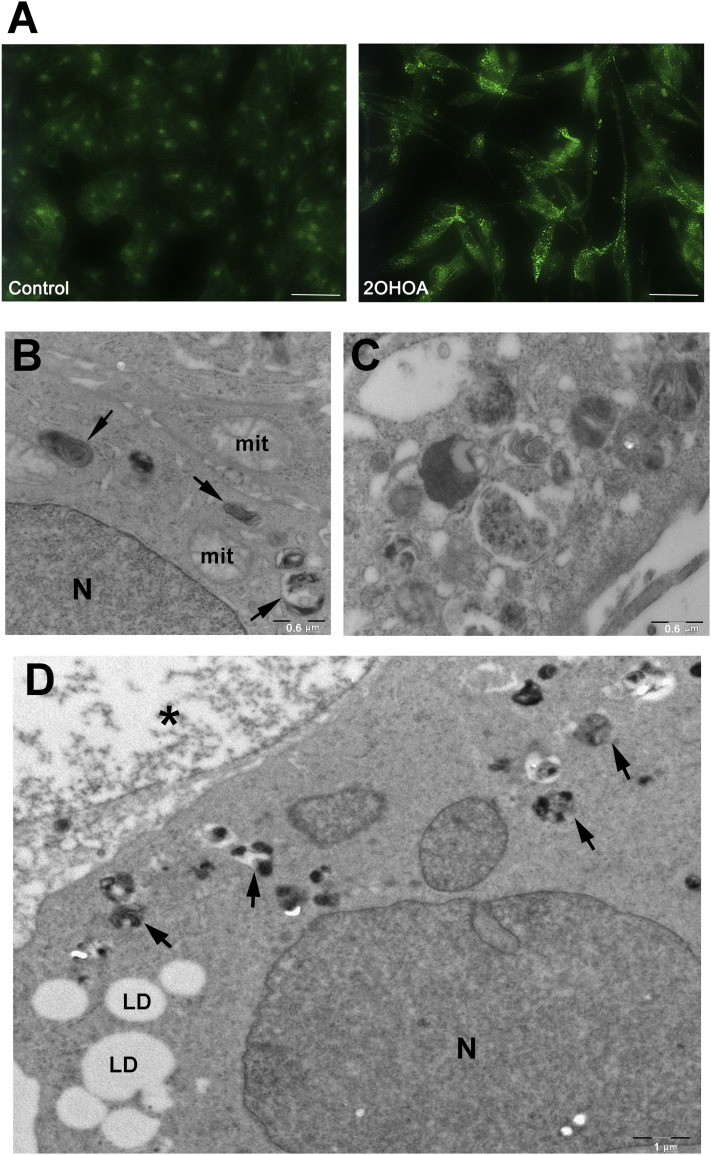
2OHOA treatment augments the number of lysosomes and induces the appearance of myelin-like bodies. (A) Representative micrographs of control and 2OHOA-treated cells (200 µM, 72 h) labeled with Lysosensor (see Experimental Procedures). Scale bar = 100 µM; (B-D) U118 cells were exposed to 2OHOA for 72 h, fixed and processed for electron microscopy. Numerous myelin-like bodies (arrows) are visible in the cell's cytoplasm (B, D). On occasion, these heterogeneous dense bodies form important aggregates, as shown in C. In addition to myelin-like bodies (arrows), lipid droplets (LD) were also observed and in the image shown, degeneration is also visible in the nucleus (*, D). N, nucleus; mit, mitochondrion.

Sphingolipids are degraded in the acidic compartments of cells, the late endosomes and lysosomes (23, 24), which we examined by staining control and treated U118 cells with Lysosensor, a probe that labels these acidic organelles. Exposure to 2OHOA (72 h, 200 µM) led to the accumulation of lysosomes (**Fig. 6A**), consistent with the increased sphingolipid mass observed at this time point. Finally, ultrastructural analyses of these U118 cells demonstrated that exposure to 2OHOA (200 µM, 72 h) induced the appearance of unidentified intracellular structures. These myelin-like bodies probably consist of accumulated sphingolipids such as SM (Fig. 6B), and they resembled the SM clusters found in several lysosomal disorders, such as Niemann-Pick disease. These bodies were distributed throughout the cytoplasm as opposed to adopting a restricted localization at cellular or nuclear membranes (Fig. 6A). At times, these myelin-like bodies tended to aggregate heterogeneously (Fig. 6B), and on occasion, small lipid droplets were also seen to concentrate in certain cytoplasmic areas (Fig. 6C).

## DISCUSSION

In this study, we demonstrate that prolonged exposure to 2OHOA induces abnormal sphingolipid accumulation and a general dysregulation of sphingolipid metabolism. This effect was accompanied by an increase in the number of lysosomes and the formation of lipid clusters similar to those observed in lysosomal storage disorders.

We previously demonstrated that the rapid and specific activation of SMS isozymes by 2OHOA is associated with a robust increase in SM mass (6). However, we did not identify the origin of the large amounts of ceramide required to sustain the increase in SM mass (ca. 150 nmol/mg protein; see Table 1 in Ref. 6). These ceramides may be derived from at least from three different pathways: *i*) de novo synthesis, *ii*) the SM cycle, or *iii*) the salvage pathway (9, 10). In the present study, we investigated each of these possibilities to determine the source of ceramides, and we concluded that all three pathways are in fact activated in glioma cells following exposure to 2OHOA.

Analysis of sphingolipids by MS revealed increases in Sph and dhSph mass, indicating the activation of both the de novo synthesis that predominantly generates dhSph and the salvage pathways, as Sph appears to be derived exclusively from the turnover of complex sphingolipids (9). As there is usually little dhSph in the cell, the large increase in its levels may be a response to the demand for sphingoid bases to act as substrates of SMS. Thus, cells may channel dhCer directly to dhSM synthesis (which increases 4.6-fold), bypassing the last step in the ceramide synthetic pathway. However, inhibition of dhCer desaturase by 2OHOA cannot be ruled out. Interestingly, both the inhibition of dhCer desaturase in human neuroblastoma (SMS-KCNR) cells (25) and exposure to 2OHOA in human lung adenocarcinoma tumor cells (1) induce cell-cycle arrest and dephosphorylation of retinoblastoma protein.

MS analysis provided further evidence of dysregulated sphingolipid metabolism, revealing an accumulation of total ceramides and an increase in HexCer mass after a 72 h exposure to 2OHOA. Interestingly, this increase was due to the selective effect of 2OHOA on C16-, C18-, and C22-containing sphingolipids, consistent with the observed increases in palmitic (16:0), stearic (18:0), and docosanoic (22:0) acids in glycerophospholipids (26). Although we observed no effect of 2OHOA on the expression of different CerS isoforms (supplementary Fig. II), an effect on the activity of these enzymes cannot be completely ruled out. The specific acyl chain moiety plays a key role in determining the final effect of a sphingolipid (27). Thus, increases in C16-ceramide are associated with the induction of apoptosis in Ramos B-cells (28), suggesting that the observed increases in this particular molecular species may also contribute to the final outcome of 2OHOA treatment (1–4, 6).

Pulse-labeling experiments provided further evidence that sphingolipid turnover increases following 2OHOA treatment, confirming the activation of de novo ceramide synthesis. In addition, the metabolic labeling of cells with NBD-sphingolipids revealed an increase in the formation of different NBD products in cells exposed to 2OHOA, indicating the breakdown and resynthesis of complex sphingolipids, such as HexCer and LacCer. These reactions occur within the acidic compartments, endosomes and lysosomes, which accumulated after 72 h exposure to 2OHOA. Finally, the formation of unidentified lamellar bodies was also associated with the accumulation of complex sphingolipids. These bodies resembled the membranous bodies formed in several sphingolipid-related disorders, such as Niemann-Pick disease, in which SMase deficiency induces the accumulation of SM (29). Similar structures are also observed when very long acyl chain ceramide synthesis is compromised (30). Consistent with the failure of 2OHOA to affect SM levels in nontumor MRC-5 cells (6), no lysosomal activation was observed after exposure to 2OHOA, confirming that these effects of 2OHOA are specific to tumor cells (2, 31).

Our results allow a putative temporal sequence of events underlying the antitumor mechanism of action of 2OHOA to be established. It should be noted that, unlike the other sphingolipids analyzed, SM levels increased significantly after shorter exposures to 2OHOA (<6 h), in agreement with the rapid activation of SMS (<5 min) (6). For this reason, we propose that the strong demand for ceramide (<200-fold of the basal ceramide level at 72 h) required to sustain the activation of SMS is the driving force that profoundly modifies both sphingolipid and phospholipid metabolism. Consequently, sphingolipid metabolism is altered to supply ceramides from all available sources, i.e., via de novo synthesis, the salvage pathway, and SM hydrolysis. This increase in SM synthesis contributes to the specific effects induced by this molecule in cancer cells, including FasR capping (3, 32) and Ras translocation from the membrane to the cytosol (2). Meanwhile, the incorporation of 2OHOA into phospholipids and its partition into membranes as a free fatty acid increase the membrane global disorder (26), which in addition to the formation of 1,2-DAG as a byproduct of the SMS reaction (2), may explain the translocation of PKC to the membrane (5, 33). As 2OHOA exposure is prolonged (<72 h), tumor cells fail to sustain this unusually high metabolism, leading to a situation resembling sphingolipidosis, whereby cell viability is compromised.

In summary, treatment with 2OHOA induces changes in the structure and composition of cancer cell membranes, effects that are not observed in noncancer cells (6). These changes promote the translocation of peripheral signaling proteins, such as PKC and Ras, which are crucial for cell growth, differentiation, and survival (2, 5). Ras translocation to the cytoplasm is followed by the inactivation of the MAPK cascade, which is involved in the overexpression of CDK inhibitors (1, 3). Consequently, the retinoblastoma protein is hypophosphorylated, producing a concomitant inhibition of the transcription factor E2F1 (necessary for cell growth and loss of differentiation) and DHFR (necessary for DNA synthesis (2, 5, 6). In addition, the PI3K/Akt pathway is inhibited following 2OHOA treatment, possibly due to inhibition of the Ras/MAPK pathway (2). This observation supports the induction of differentiation by 2OHOA treatment, as both the MAPK and Akt pathways induce a loss of differentiation in cancer cells, and it in part explains the observed induction of autophagy caused by p27^kip1^ activation and Akt inhibition (34). Taken together, the present findings provide important insights into the key events in the specific regulation of cancer cell lipid metabolism. The dramatic changes in sphingolipid levels described here are likely to reflect the specific activation of SMS following 2OHOA treatment, which may in turn explain the specific induction of cancer cell death.

## Supplementary Material

Supplemental Data
