# Supplementary material for: Sustained activation of sphingomyelin synthase by 2-hydroxyoleic acid induces sphingolipidosis in tumor cells
Source: J Lipid Res. 2013 May;54(5):1457–65. doi: 10.1194/jlr.M036749 (PMC3653406; doi:10.1194/jlr.M036749)
Supplement: Supplemental Data [file supp_54_5_1457__index.html]

The sustained activation of sphingomyelin synthase by 2-hydroxyoleic acid induces sphingolipidosis in tumor cells — Sustained activation of sphingomyelin synthase by 2-hydroxyoleic acid induces sphingolipidosis in tumor cells — Supplemental Data 

# Sustained activation of sphingomyelin synthase by 2-hydroxyoleic acid induces sphingolipidosis in tumor cells

## Supplemental Data

**Files in this Data Supplement:**

- Supplementary Information - Table S1. CerS primers used in this study Figure S1. CerS mRNA levels in U118 cells. Figure S2. 2OHOA alters the turnover of sphingolipids A549 cells. Materials and Methods Quantitative Reverse Transcription-Polymerase Chain Reaction (QRT-CR)
