# Supplementary material for: Sustained activation of sphingomyelin synthase by 2-hydroxyoleic acid induces sphingolipidosis in tumor cells
Source: J Lipid Res. 2013 May;54(5):1457–65. doi: 10.1194/jlr.M036749 (PMC3653406; doi:10.1194/jlr.M036749)
Supplement: Supplemental Data [file supp_M036749_jlr.M036749-1.pdf]

The sustained activation of sphingomyelin synthase by 2-hydroxyoleic acid induces *sphingolipidosis* in tumor cells

Maria Laura Martin[*], Gerhard Liebisch[†], Stefan Lehneis[†], Gerd Schmitz[†], María Alonso-Sande[*], Joan Bestard-Escalas[*], Daniel H. Lopez[*], José Manuel García-Verdugo[§], Mario Soriano-Navarro[§], Xavier Busquets[*], Pablo V. Escribá[*] and Gwendolyn Barceló-Coblijn[*, ˥]

*Laboratory of Molecular Cell Biomedicine, Department of Biology, IUNICS, University of the Balearic Islands, Ctra. de Valldemossa Km 7.5, E-07122 Palma, Balearic Islands, Spain.

†University of Regensburg, Institute for Clinical Chemistry and Laboratory Medicine, Franz-Josef-Strauss-Allee 11, 93042 Regensburg, Germany.

§Laboratorio de Morfología Celular, Unidad Mixta CIPF-UVEG, CIBERNED, 46013 Valencia, Spain.

Running title: 2-hydroxyoleic acid induces sphingolipidosis

˥ Corresponding author:

Laboratory of Molecular Cell Biomedicine, Department of Biology, University of the Balearic Islands, Ctra. de Valldemossa Km 7.5, E-07122 Palma, Balearic Islands, Spain. Tel.: +34 971259782; Fax: +34 971173184; Email: gwendolyn.barcelo@uib.cat

Table 1. CerS primers used in this study

Gene	Orientation	Sequence (5’-3’)
CerS1	Forward	GGC CAC TCC ATC TAC GCT AC
	Reverse	ACT CAA GCT GCA CGT CAC TG
CerS2	Forward	TCT CTA TAT CAC GCT GCC CC
	Reverse	GCT TGC CAC TGG TCA GGT AG
CerS3	Forward	ATC AAG AGA GGC CTT CCA GG
	Reverse	ATA GCC ATT CCA AAC CTC CC
CerS4	Forward	ATC AGA CCA GGA GGC AAG TG
	Reverse	GAA CTT CTT GGT CAG CTG GG
CerS5	Forward	CCA ATG CCA TCC TTG AAA AG
	Reverse	AAC CAG CAT TGG ATT TTT CG
CerS6	Forward	TTC GAC AAA GAC GCA ATC AG
	Reverse	AGC AAT GCC TCG TAT TCC AC
β-actin	Forward	GCG GGA AATCGT GCG TGA CAT
	Reverse	CTA CCT CAACTT CCA TCA AAG CAC

Figure S1.

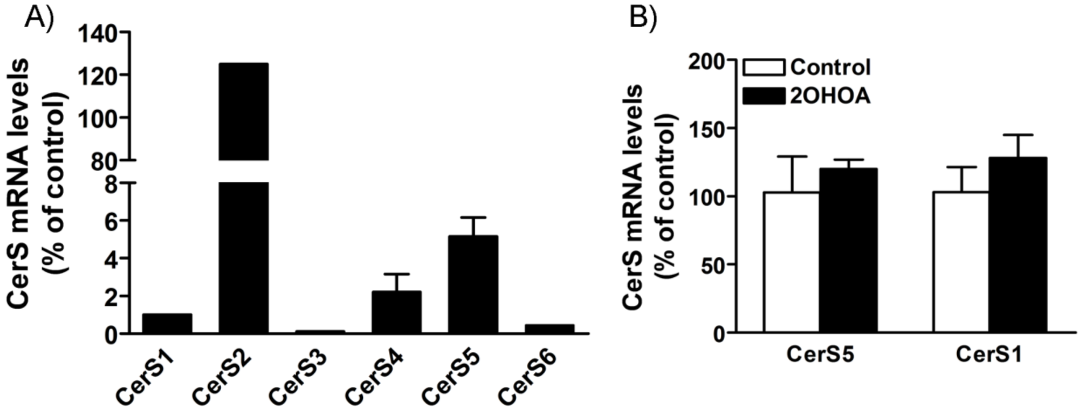

Figure S1. CerS mRNA levels in U118 cells. (A) RNA was extracted from U118 control (untreated) cells and the expression of different CerS mRNAs were analyzed by qRT-PCR as described in the Experimental Procedures. Consistent with previous findings (23), CerS2 was the most abundant CerS isoform expressed in U118 cells (Fig. 2A), a CerS isoform that synthesizes mainly C20-26 species consistent with the sphingolipid fatty acid profile previously described. CerS5 and CerS4 expression, which synthesize C16 and C18-C20, respectively, was weaker than that of CerS2 (Fig. 2A), while CerS1 and CerS6 expression was very weak and CerS3 was almost undetectable, in agreement with the strong tissue specificity of the latter for testis and skin (24). (B) Effect of 2OHOA on CerS1 and CerS5 mRNA expression. U118 cells were incubated in the presence (200 µM, 72 h) or absence (control) of 2OHOA. The values are expressed as the percentage of the control group and represent the mean ± SEM (n = 4).

Figure S2.

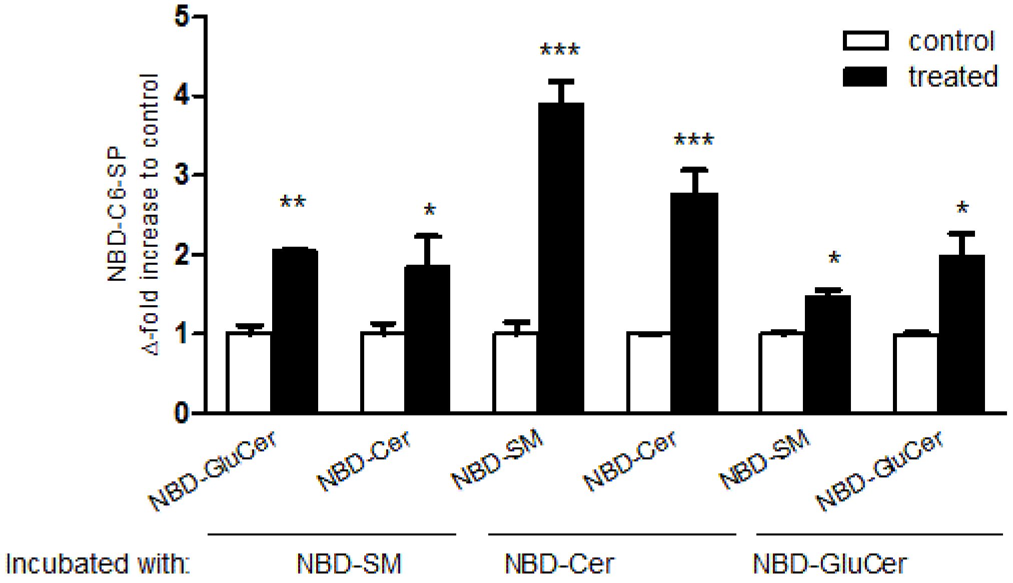

Figure S2. 2OHOA alters the turnover of sphingolipids A549 cells. Control and 2OHOA-treated cells (200 µM, 24 h) incubated with NBD-Cer, NBD-SM or NBD-GlcCer (3 µM, 3 h), from which lipids were extracted and analyzed by TLC. NBD-C6-sphingolipids (NBD-C6-SP) were quantified as described in Material and methods section. The values represent the mean ± SEM (n = 3) and the asterisks (*) indicate a significant effect of treatment as compared with controls: *P < 0.05; **P < 0.01; ***P < 0.001.

Materials and Methods

Quantitative Reverse Transcription-Polymerase Chain Reaction (QRT-CR) U118 cells were incubated in the presence or absence of 2OHOA for 24 h before their total RNA was extracted using the RNeasy Mini kit and any residual DNA was removed with the RNase-free DNase kit (Qiagen), according to the manufacturer's instructions. The total amount and purity of the RNA was determined using a Nanodrop 1000 spectrophotometer (Thermofisher Scientific, Waltham, MA), measuring the optical density at 260 and 280 nm. Product integrity was assessed by electrophoresis on a 1% agarose gel stained with ethidium bromide. RNA was reverse transcribed in a thermal cycler (Eppendorf Master Gradient) using the Transcriptor First Strand cDNA Synthesis Kit (Roche Diagnostics), according to the manufacturer's instructions. For PCR amplification, primers were designed using the SDSC Biology Workbench Program (http://workbench.sdsc.edu) based on gene sequences from GenBank. To differentiate between cDNA and genomic DNA, the primers were designed to target distinct sites of the exon-exon boundaries (Table 1). Real-time PCR amplifications were carried out in 96-well plates in a StepOne Plus thermal cycler (Applied Biosystems) using the SYBR Premix Ex Taq 2× (Perfect Real Time, Takara), containing an internal ROX 1 probe and 0.1 µM of each primer. An initial denaturation step at 95°C for 30 s preceded thermal cycling. DNA amplification and fluorescence quantification was determined over 35 cycles with a denaturation step at 95°C for 5 s, followed by an annealing/extension step at 60°C for 34 s. Finally, fluorescence detection and quantification was carried out after each DNA extension step, and the data were analyzed using StepOne software (v2.0) using β-actin expression as an endogenous control as this is not modulated by 2OHOA. The ratio between the expression of the genes of interest and that of β-actin was determined using the equation described by Pfaffl and coworkers {Pfaffl, 2001 #170}, and the efficiency of the reaction was estimated by the increase in absolute fluorescence, according to Pfaffl (unpublished observations; http://www.gene-quantification.de/efficiency01.html#e-fluoro-2). Melting curve analysis and agarose gel electrophoresis were used to further characterize the PCR products.
